# *Escherichia coli* from urine samples of pregnant women as an indicator for antimicrobial resistance in the community: a field study from rural Burkina Faso

**DOI:** 10.1186/s13756-022-01142-7

**Published:** 2022-09-05

**Authors:** Annelies S. Post, I. Guiraud, M. Peeters, P. Lompo, S. Ombelet, I. Karama, S. Yougbaré, Z. Garba, E. Rouamba, H. Tinto, Jan Jacobs

**Affiliations:** 1 Department of Internal Medicine, Radboud Centre for Infectious Diseases, Radboud University Medical Centre, Nijmegen, The Netherlands; 2 Unit of Tropical Laboratory Medicine, Department of Clinical Sciences, Institute of Tropical Medicine, Antwerp, Belgium; 3 IRSS/Clinical Research Unit of Nanoro (CRUN), Nanoro, Burkina Faso; 4 Department of Microbiology, Immunology and Transplantation, KU Leuven, Leuven, Belgium; 5 Institut Supérieur des Sciences de la Santé, Université Nazi Boni de Bobo-Dioulasso, Bobo-Dioulasso, Burkina Faso

**Keywords:** Antimicrobial resistance, Community, Asymptomatic bacteriuria, ANC, Pregnancy, *Escherichia coli*, Rural Africa, Burkina Faso

## Abstract

**Background:**

In low- and middle-income countries, surveillance of antimicrobial resistance (AMR) is mostly hospital-based and, in view of poor access to clinical microbiology, biased to more resistant pathogens. We aimed to assess AMR among *Escherichia coli* isolates obtained from urine cultures of pregnant women as an indicator for community AMR and compared the AMR results with those from *E. coli* isolates obtained from febrile patients in previously published clinical surveillance studies conducted within the same population in Nanoro, rural Burkina Faso. We furthermore explored feasibility of adding urine culture to standard antenatal care in a rural sub-Saharan African setting.

**Methods:**

Between October 2016–September 2018, midstream urine samples collected as part of routine antenatal care in Nanoro district were cultured by a dipslide method and screened for antibiotic residues. Significant growth was defined as a pure culture of *Enterobacterales* at counts of ≥ 10^4^ colony forming units/ml.

**Results:**

Significant growth was observed in 202/5934 (3.4%) cultures; *E. coli* represented 155 (76.7%) of isolates. Among *E. coli* isolates, resistance rates to ampicillin, cotrimoxazole and ciprofloxacin were respectively 65.8%, 64.4% 16.2%, compared to 89.5%, 89.5% and 62.5% among *E. coli* from clinical isolates (n = 48 of which 45 from blood cultures). Proportions of extended spectrum beta-lactamase producers and multidrug resistance were 3.2% and 5.2% among *E. coli* isolates from urine in pregnant women versus 35.4%, and 60.4% respectively among clinical isolates.

**Conclusions:**

The *E. coli* isolates obtained from healthy pregnant women had significantly lower AMR rates compared to clinical *E. coli* isolates, probably reflecting the lower antibiotic pressure in the pregnant women population. Adding urine culture to the routine urine analysis (dipstick) of antenatal care was feasible. The dipslide culture method was affordable and user-friendly and allowed on-site inoculation and easy transport; challenges were contamination (midstream urine sampling) and the semi-quantitative reading. Provided confirmation of the present findings in other settings, *E. coli* from urine samples in pregnant women may be a potential indicator for benchmarking, comparing, and monitoring community AMR rates across populations over different countries and regions.

**Supplementary Information:**

The online version contains supplementary material available at 10.1186/s13756-022-01142-7.

## Introduction

Antimicrobial resistance (AMR) rises globally and is a threat to public health, particularly in low- and middle-income countries (LMIC) [1]. Surveillance is one of the five domains of the World Health Organization's Global Action plan against AMR [2]. The Global Antimicrobial Resistance Surveillance System (GLASS) set outs standards for collection, analysis and sharing of AMR data at a worldwide level. Aggregated surveillance data reported to GLASS rely on antibiotic susceptibility testing (AST) results of clinical samples [3].

However, surveillance of clinical samples has shortcomings particularly when applied in LMIC. First, LMIC face problems of access to competent and quality-assured clinical bacteriology [4]. As a result, samples processed in LMIC settings may be biased to more advanced disease stages and collected under coverage of empiric antibiotic treatment. Second, surveillance by clinical samples may be influenced by the type of clinical specimen (e.g. blood vs. respiratory tract secretions), previous antibiotic use as well as indications for sampling, factors that are often not standardized in LMIC [5]. Finally, clinical bacteriology in LMIC is typically implemented at the second level of care, i.e. the district referral hospital [6]. Although samples may be collected at the primary level of care (health post, health center), expertise and skills for sampling as well as reliable transport systems may be lacking [1]. By consequence, the deducted surveillance data may tend towards an overestimation of AMR rates and may not reflect AMR rates at community level.

Using *Escherichia coli* isolates obtained from pregnant women could be a way to circumvent these shortcomings. First, urine is already routinely collected at antenatal care visits at all healthcare levels throughout LMIC. Second, the collection method and specimen are universally comparable. Third, pregnant women tend to use fewer antibiotics (for concerns of safety for the unborn child), which means that there is a relatively low antibiotic pressure among this population compared to patients. And finally, urine cultures, once inoculated during ANC visit, can be transported to a larger healthcare facility with microbiology lab with minimal effect on the reliability of the culture result.

The primary objective of this study was to assess if *E. coli* isolates recovered from urine samples of healthy pregnant women can serve as a proxy for AMR surveillance at the community level in rural West-Africa. We focused on *E. coli* as it is the most frequent isolate in asymptomatic bacteriuria in pregnancy [7]. In addition, *E. coli* is a key pathogen in current programs that monitor AMR in humans [3] and in One Health populations (AGISAR) [8]. Our secondary objective was to explore whether adding bacteriological culture of urine to the routine antenatal care is feasible in a rural West-African setting.

## Methods

### Study design

We conducted a cross-sectional study recruiting pregnant women attending routine antenatal care (ANC) in rural Burkina Faso. Urine samples routinely obtained for dipstick analysis (glucose and protein) were semi-quantitatively cultured by dipslide technique to assess for asymptomatic bacteriuria (ASB). For the purpose of this study, isolates growing in counts of ≥ 10^4^ colony forming units (CFU/ml) belonging to the *Enterobacterales* species and *Enterococcus faecalis* were considered as "significant growth". Antibiotic susceptibility testing was done for *E. coli* isolates. AMR profiles were compared to *E. coli* isolates obtained from blood culture surveillance studies performed in the same district and the same laboratory. Women were asked about recent antibiotic use prior to sampling and urine samples were screened for antibiotic residues.

### Study site, period and participants, routine antenatal care

**Fig. 1:**
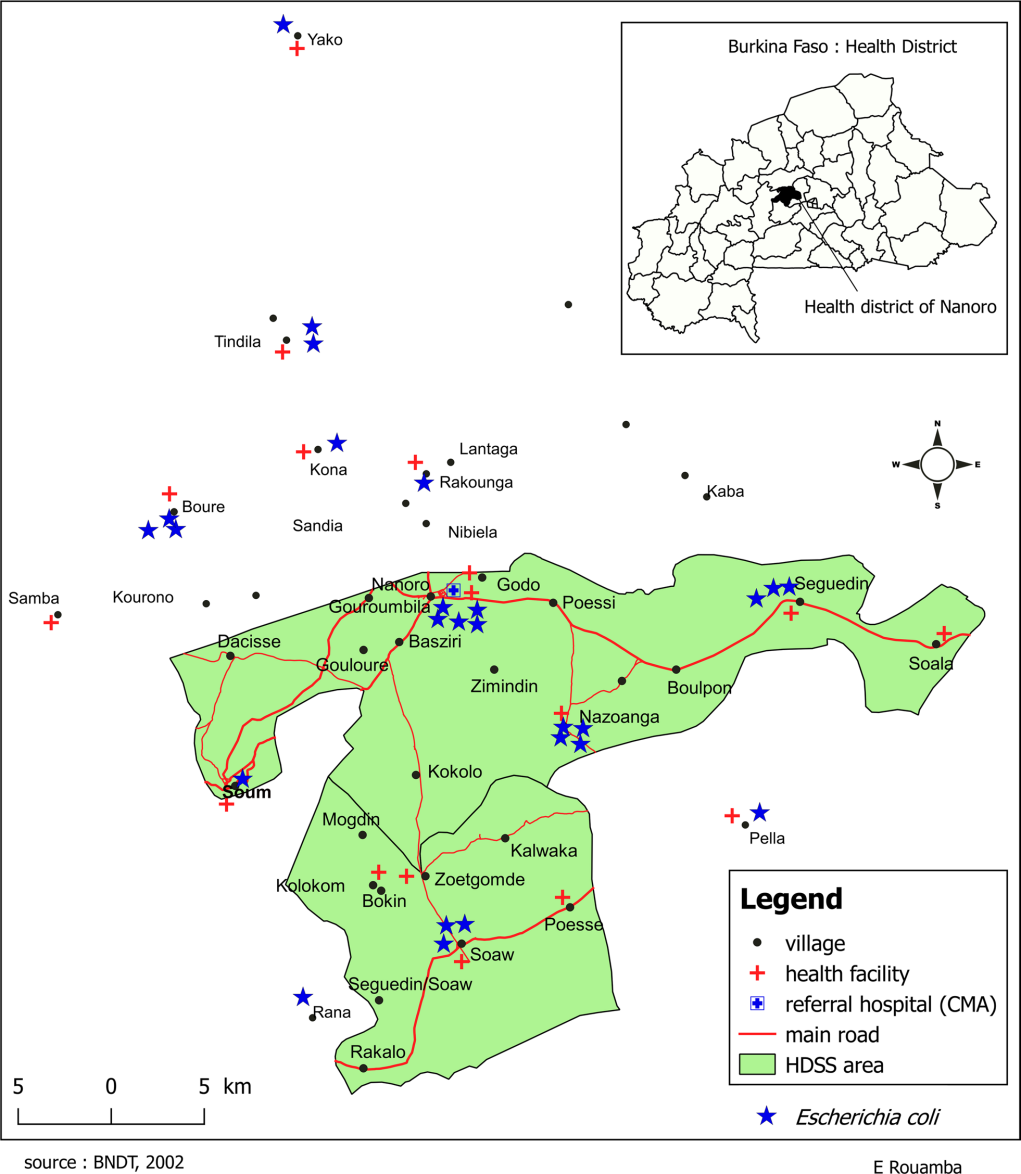
Map of the Health and Demographic Surveillance Site of Nanoro. The blue stars indicate residences of women with cultured *E. coli*. There were no evident clusters. Cases were living within and just outside the health and demographic surveillance site

The study was conducted from October 2016 to September 2018 at the Clinical Research Unit of Nanoro (CRUN) in the Center-West Region of Burkina Faso. Samples were obtained in 9 health centers within the Health and Demographic Surveillance System (HDSS) of CRUN, at 11–38 km from CRUN (Fig. 1). The HDSS monitors changes in a total population of 60,000 persons distributed over 24 villages [9]. Routine ANC is provided at the health centers and is organized in morning hours between 7 and 12 a.m. A total of 4 ANC visits are recommended during each pregnancy [10] and comprise collection of demographic data (age, week of pregnancy and Gestation Parity Abortion score [GPA score]) and uranalysis for glucose and protein by dipstick test.

### Study intervention: urine culture and collection of demographic and clinical data

Informed consent was sought from all women attending routine ANC by the ANC nurse. After written consent was obtained, a study nurse provided a sterile cup and instructions on how to obtain a clean midstream urine sample. Apart from routinely collected ANC data, information on antibiotic use in the past 2 weeks, and signs and symptoms of urinary tract infection were collected. For semi-quantitative culture, dipslide devices (Uricult MC/CLED, International Medical Products, Brussels, Belgium and Servocults, Meus S.R.L, Padova, Italy) were used. The dipslide consisted of cysteine-, lactose, and electrolyte-deficient (CLED) agar on one side and MacConkey agar on the other side. Inoculation was done by the study nurse: dipslides were fully submerged in the urine sample, alternatively, a sterile pipette was used to inoculate both agar slides. The dipslide was then placed straight-up on a piece of absorbent paper to allow excess urine to leak off. Subsequently, the urine was tested for presence of glucose and protein using a dipstick analysis (UroColor strips [Standard Diagnostics, Gyeonggi-do, Republic of Korea] or Urine-10 strips [Cypress Diagnostics, Hulshout, Belgium]). Leukocyturia was quantified according to the manufacturers’ instruction as negative (−), + (25–74 cells/µL), ++ (75–499 cells/µL), or +++ (500 or more cells/µL). The urine dipslides and the left-over urine samples were stored in the fridge (2–8 °C). Transport to the laboratory was done in a light protected box by motorcycle, within 24 h after collection, at room temperature.

### Semi-quantitative culture, bacterial identification and antibiotic susceptibility testing

Upon reception at the laboratory of CRUN, dipslides were incubated for 16–24 h at 35 °C. Grown cultures were assessed for colony counts by comparing the number of colonies to the figure provided in the product's instructions for use. Bacterial isolates were identified using standardized biochemical techniques and API (bioMérieux, Marcy l’Etoile, France) in case of doubtful test reactions. The isolates were subsequently stored in Tryptic Soy Agar (CM0131, Oxoid Ltd). All bacteria growing in counts of ≥ 10^4^ colony forming units/ml (CFU/ml) except for non-fermentative Gram-negative bacteria and bacteria considered as contaminants [*Bacillus* spp. and coagulase-negative staphylococci (CNS)] were shipped to the Institute of Tropical Medicine in Antwerp (Belgium) for confirmation of identification by matrix-assisted laser desorption/ionization time-of-flight (MALDI-TOF) technology (Bruker MALDI Biotyper, Bruker, Billerica, MA, US) at the University Hospital of Leuven (Belgium). Isolates identified as *E. coli* were processed for AST by disk diffusion (Neo-Sensitabs, Rosco Diagnostica A/S, Taastrup, Denmark) according to Clinical and Laboratory Standards Institute (CLSI) guidelines [11]. Combination disk tests (Neo-Sensitabs, Rosco Diagnostica A/S, Taastrup, Denmark) were performed to assess the production of Extended Spectrum ß-lactamases (ESBL) [11]. Neo-Sensitabs disk contents were as follows: nitrofurantoin 300 µg, fosfomycin 200 µg, ampicillin 10 µg, cotrimoxazole (trimethoprim 1.25 µg + sulfamethoxazole 23.75 µg), ciprofloxacin 5 µg, gentamicin 10 µg, ceftriaxone 30 µg, ceftazidime 30 µg, ceftriaxone-clavulanic acid 30 + 10 µg and ceftazidime-clavulanic acid 30 + 10 µg.

### Antibiotic residue testing

Urine samples were tested for the presence of antibiotic residues as part of the work-up at the laboratory of CRUN. For each urine sample, a Mueller–Hinton plate was inoculated with 0.5 McFarland saline solutions of *Bacillus spizizenii* (ATCC 6633). An absorbent paper disk was saturated with urine and placed on the Mueller–Hinton agar. Plates were incubated at 35 °C for 18–24 h. The appearance of an inhibition zone around the urine disk was considered positive for the presence of residue antibiotics [12].

### Comparison with clinical isolates

For comparison, AST results from clinical isolates of urine and blood cultures collected under different study protocols in the Nanoro district hospital and processed at CRUN during the same period were used. Two studies assessed the proportion and differentiation of malaria and bacteremia in the Nanoro district hospital [13, 14], a third study assessed the incidence and reservoir of non-typhoidal *Salmonella* bloodstream infection [15, 16] and finally, several isolates were obtained from a blood culture surveillance study for follow-up of antimicrobial resistance (unpublished data).

### Definitions

For the purpose for this study, single-organism cultures with counts of ≥ 10^4^ CFU/ml belonging to the *Enterobacterales* species or *Enterococcus faecalis* were considered as "significant growth" and the isolates were considered as “pathogens”. Isolates obtained from febrile patients are referred to as “clinical samples”. Asymptomatic bacteriuria was defined as the presence of actively multiplying bacteria in the urinary tract in patients that have no obvious symptoms of urinary tract infection (UTI) [17]. Skin- or environmental bacteria (CNS,* Bacillus* spp*.*), non-fermentative Gram-negative bacteria and bacteria growing as mixed flora (≥ 2 different isolates) were considered as contaminants [12]. In case a culture grew with mixed isolates including *Enterobacterales*, the latter were also subcultured for the purpose of antibiotic susceptibility testing (AST). *Staphylococcus aureus* isolates were not considered for antibiotic susceptibility testing.

Multi-drug resistance (MDR) for *Enterobacterales* was defined as resistance to the three principal oral antibiotic categories for urinary tract infection (penicillins, cotrimoxazole and fluoroquinolones). The number of parities of each participant was categorized as in nullipara (never given birth), primipara (given birth once), multipara (≥ 2 births) or grand multipara (≥ 5 births) [18].

### Sample size, data registration and statistical analysis

In line with the CLSI M39 [19], a minimum number of 30 *E. coli* isolates was targeted for separate antibiotic susceptibility reporting. Assuming a prevalence of 5–10% asymptomatic bacteriuria with 10% contamination rate and *E. coli* being 40% of retrieved isolates, 6000 women were targeted. Data were recorded in a coded database (Microsoft Excel, Redmond, US). Differences in proportions were compared using as appropriate a Mann–Whitney-u test, a Kruskall Wallis test or a Chi-square test. For smaller sample sizes (value in one of the cells ≤ 5), the Fischer exact test was used. A *p* value of 0.05 was considered as statistically significant. Reporting of the methods and results was done according to the STROBE guidelines for cross-sectional studies [20].

### Ethics

The study was approved by the national ethics committee of Burkina Faso (Comité d’Ethique pour la Recherche en Santé (Reference No. 2015-7-96 July 1st, 2015), the institutional review board of ITM, Antwerp (Reference 1008/15 from December 15th, 2015) and the ethics committee of the University Hospital of Antwerp (Reference 15/51/563, January 4th, 2016). Written informed consent was obtained before participation in the study. A screening log with reasons for refusal was completed at each health center included in the study. If ASB was diagnosed, laboratory staff of the study site communicated the recovery of clinically significant isolates and their AST results to the study investigator, who informed the ANC nurse or the clinician responsible of the ANC. Participants were treated according to national treatment guidelines.

## Results

### Characteristics of study participants

Over a time-period of 2 years (October 2016 to September 2018) a total of 6018 urine samples were collected. A total of 84 (1.4%) samples were excluded from analysis because the participant reported symptoms suggestive of urinary tract infection, leaving 5934 samples representing 5907 unique participants (27 participants were sampled on two separate ANC visits). Secondary analyses concerning parity and dipstick analyses and prior antibiotic use were done on samples for which data were complete (n = 5741 [96.7%]). A cluster of missing data included 36 consecutive dipstick analyses obtained between June 11th and August 8th from Healthcare centre Seguedin.

The age of participants for whom full data were available ranged from 14 to 50 years. Overall, the median (interquartile range [IQR]) age was 25 (20–30) years. In total 23.9% of samples were obtained from nulli- or primipara. All other cases were multi (43.4%) or grand multipara (32.6%) (Additional file 1: Table S1). Most samples were obtained from women in the second (32.9%) or third (63.1%) trimester of pregnancy (Table 1).

**Table 1:** Total numbers of samples with numbers of significant growth for 5741 healthy pregnant women, matched by parity and trimester of pregnancy

Patients for whom complete data were available	Total		Trimester 1		Trimester 2		Trimester 3	
n = 5741	n = 5741		n = 227		n = 1889		n = 3625	
	nr. Cases	% growth	nr. Cases	% growth	nr. Cases	% growth	nr. Cases	% growth
*Gestation*								
Nullipara (percentage significant growth)	7	0%	1	0%	1	0%	5	0%
Primipara (percentage significant growth)	1367	3.9%	65	4.6%	538	3.9%	784	3.7%
Multipara (percentage significant growth)	2493	3.0%	99	4.0%	832	3.5%	1562	2.7%
Grand multipara (percentage significant growth)	1874	2.4%	62	0%	518	2.9%	1294	2.3%
*Age distribution (years [IQR])*	25 (20–30)		24 (20–30)		24 (19–30)		26 (21–30)	
*Dipstick results*								
Nitrite* (n [%])	68 (1.2%)	7 (3.1%)	29 (1.5%)				32 (0.9%)	
Leukocytes* (n [%])	1055 (18.4%)	44 (19.4%)	318 (16.8%)				717 (19.1%)	
Dipslide results**								
< 10^4^ CFU/ml	2865 (49.9%)		125 (55.1%)		905 (47.9%)		1835 (50.6%)	
≥ 10^4^ CFU/ml	643 (11.2%)		18 (7.9%)		184 (9.7%)		441 (12.1%)	
Clinically significant growth	173 (3.4%)		7 (3.1%)		65 (3.4%)		101 (2.8%)	
Asymptomatic bacteriuria	97 (1.7%)		4 (1.8%)		37 (2.0%)		56 (1.5%)	

Significant growth is defined as growth of *Enterobacterales* in counts of ≥ 10^4^ CFU/ml. Parity was defined as follows: nullipara = never give birth, primipara = given birth once, multipara = parity ≥ 2, grand multipara = parity ≥ 5 [18]

**Fig. 2:**
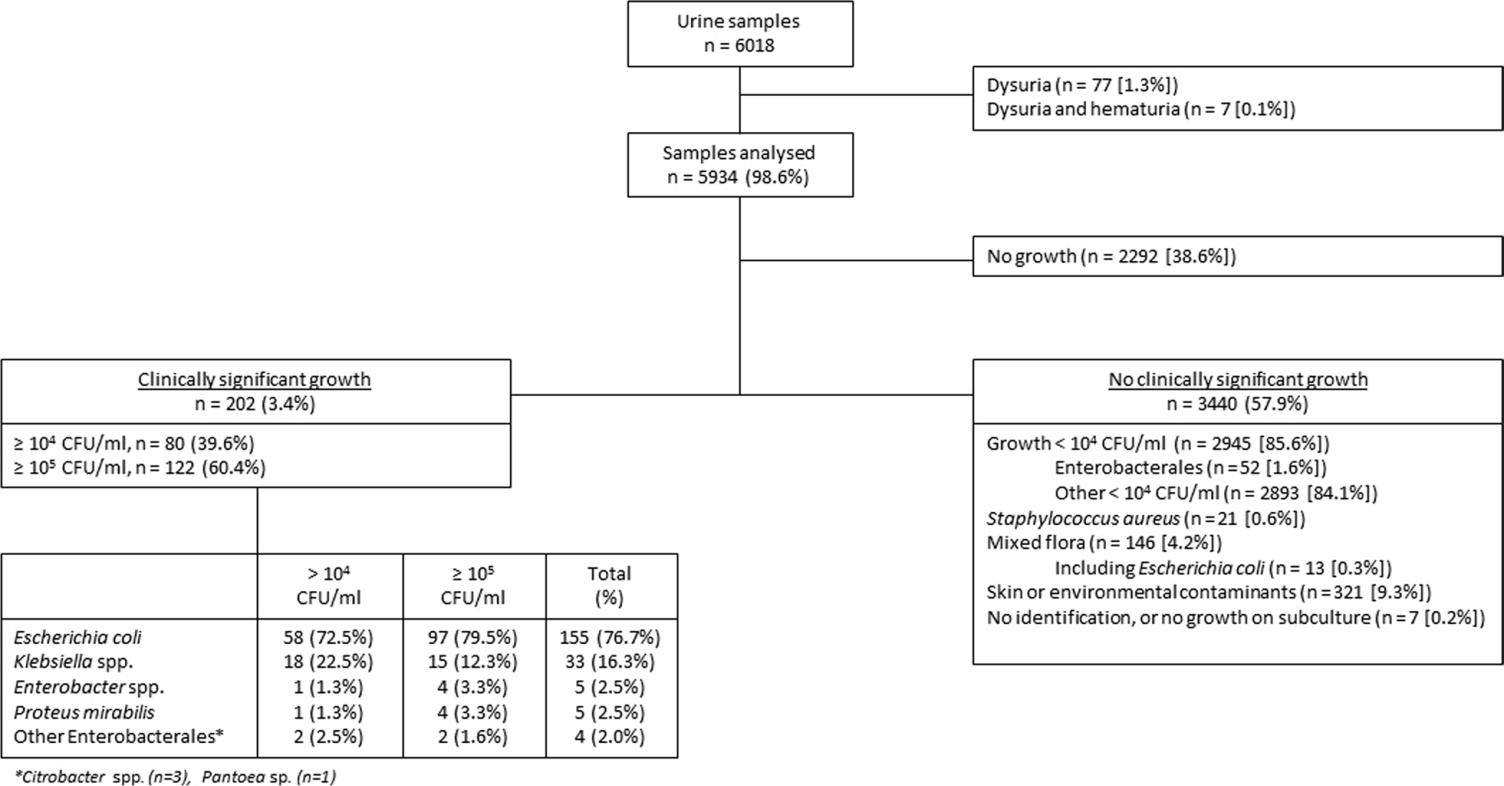
Clinically significant growth was defined as isolates growing in counts of ≥ 10^4^ CFU/ml belonging to the Enterobacterales species or *Enterococcus faecalis*. Clinically significant isolates growing in counts of ≥ 10^5^ CFU/ml were defined as ASB

Data differ slightly from those in Fig. 2 where data are presented for all samples

Clinically significant growth is defined as growth with an *Enterobacterales* species in counts of ≥ 10^4^ CFU/ml

Asymptomatic bacteriuria is defined as clinically significant growth with counts of ≥ 10^5^ CFU/ml

### Breakdown of samples, proportions of significant growth, species recovered

A breakdown of samples and significant growth is presented in Fig. 2. In total 2292 (38.6%) of dipslides did not have any growth, 2945 (49.6%) had growth of < 10^4^ CFU/ml and 697 (11.6%) had growth of ≥ 10^4^ CFU/ml. In total 202 samples (28.9% of grown cultures and 3.4% of all samples) showed significant growth (growth ≥ 10^4^ CFU/ml), of which 122 (2.1%) reached ≥ 10^5^ CFU/ml and therefore qualified as ASB.

*Escherichia coli* accounted for 76.7% of pathogens obtained (155/202) from cultures with ≥ 10^4^ CFU/ml. Other species were *Klebsiella* spp. (n = 32 [15.8%]), *Proteus mirabilis* (n = 5 [2.5%]), *Enterobacter* spp. (n = 5 [2.5%]), *Citrobacter* spp. (n = 3 [1.5%]) and *Pantoea* (n = 1 [0.5%]). Species distribution between samples with ≥ 10^4^ CFU/ml and ≥ 10^5^ CFU/ml did not differ significantly (Mann–Whitney *p* < 0.001).

### *Escherichia coli* from clinical samples

A total of 48 *E. coli* isolates were obtained from clinical samples in the Nanoro district hospital between 2012 and 2019. Forty-five isolates were retrieved from blood cultures and 3 isolates from urine cultures. None of the isolates from clinical samples were obtained from pregnant women.

### Antibiotic resistance among *E. coli* from healthy pregnant women and febrile patients

AMR profiles of both the clinical isolates and isolates from healthy pregnant women are presented in Table 2. Resistance to ampicillin, cotrimoxazole and ciprofloxacin were respectively 65.8%, 64.4% 16.2%. MDR was observed in 5.2% isolates; resistance to gentamicin occurred in 3.9% isolates. There were 5 (3.2%) ESBL producing *E. coli* isolates; 2 of which were co-resistant to cotrimoxazole and ciprofloxacin, of which one was co-resistant to gentamicin as well. There was no resistance to fosfomycin and only 3.9% resistance to nitrofurantoin among *E. coli* recovered from urine in healthy pregnant women.

**Table 2:** Proportions of antibiotic resistance and combined antibiotic resistance for *Escherichia coli* obtained from urine samples of healthy pregnant women compared to clinical samples from febrile patients

Resistance per antibiotic	Healthy pregnant women	Febrile patients
	Urine culture (n = 155)	Urine culture (n = 3) blood culture (n = 45)
	n (%)	n = 48 (%)
Nitrofurantoin	6 (3.9%)	Not done
Fosfomycin	0 (0%)	Not done
Ampicillin	102 (65.8%)	43 (89.6%)
Cotrimoxazole	97 (64.4%)	43 (89.6%)
Ciprofloxacin	25 (16.2%)	30 (62.5%)
Gentamicin	6 (3.9%)	11 (22.9%)
Ceftriaxone	5 (3.2%)	18 (37.5%)
ESBL producers	5 (3.2%)	17 (35.4%)

Combined resistance	n (%)	n (%)
Ampicillin + cotrimoxazole	80 (51.6%)	41 (85.4%)
Ampicillin + cotrimoxazole + ciprofloxacin	8 (5.2%)	29 (60.4%)
Ampicillin + cotrimoxazole + gentamicin	3 (1.9%)	11 (22.9%)
Ampicillin + cotrimoxazole + ciprofloxacin + gentamicin	2 (1.3%)	11 (22.9%)
ESBL + cotrimoxazole	2 (1.3%)	17 (35.4%)
ESBL + cotrimoxazole + ciprofloxacin	2 (1.3%)	16 (33.3%)
ESBL + cotrimoxazole + gentamicin	1 (0.6%)	7 (14.5%)
ESBL + cotrimoxazole + ciprofloxacin + gentamicin	1 (0.6%)	7 (14.5%)

Differences in proportions of clinical and urine isolates were assessed using chi-square test. For smaller sample sizes (value in one of the cells ≤ 5), the Fischer exact test was used. All differences between isolates obtained from healthy pregnant women and febrile patients were statistically significant (*p* < 0.001)

There was no statistical difference in resistance patterns between isolates growing in counts of 10^4^ CFU/ml and 10^5^ CFU/ml

In contrast, resistance rates among isolates obtained from febrile patients were significantly higher for all individual antibiotics as well as combinations. Out of 48 isolates, 43 (89.5%) were resistant to each ampicillin and cotrimoxazole, and 30 (62.5%) were resistant to ciprofloxacin; MDR was observed in 60.4% of isolates. Resistance to gentamicin was lowest at 22.9%. In total 18 isolates showed resistance to ceftriaxone; all but one (35.4% of total) were confirmed as ESBL producers. All ESBL producing isolates were co-resistant to cotrimoxazole and all but one were co-resistant to ciprofloxacin.

Additional file 1: Table S2 shows an overview of antibiotic susceptibility profiles of other major pathogens obtained from healthy pregnant women; other *Enterobacterales* growing in ≥ 10^4^ CFU/ml, *E. coli* obtained from urine samples growing in mixed flora. This overview shows that antibiotic resistance among other potential pathogens (i.e. *Klebsiella* spp*.*, *Enterobacter* spp., *Proteus* spp.) was also relatively low. One *Klebsiella pneumoniae* and one *Enterobacter cloacae* were ESBL-producers (2/44, 4.5% of non-*E. coli Enterobacterales*).

### Trimester and parity

Most (120/202 [59.4%]) participants with significant growth were in their third trimester of pregnancy, differences with the first and second trimester were however not significant (Kruskall Wallis, *p* = 0.6). There was also no relation between proportion of significant growth and parity (Kruskall Wallis, *p* = 0.07).

### Antibiotic use prior to sampling

In total 96 (1.7%) samples screened positive for antibiotic residue. Previous antibiotic use was reported by 29 patients, of whom 2 had antibiotic residues in their urine samples. One participant with significant growth reported antibiotic use prior to sampling and four participants screened positive for antibiotic residues.

### Leukocyte esterase and nitrite

Leukocyturia was present in 1084 (18.4%) samples and nitrite in 74 (1.2%) samples. For detection of significant growth, the positive predictive and negative predictive values of leukocyturia were 6.7% and 97.3% respectively; for nitrite they were 37.8% and 97.0% and for leukocyturia and nitrite combined they were 48.7% and 96.9% (Additional file 1: Table S3).

## Discussion

### Summary of findings

The present study assessed the AMR rates of *E. coli* present as significant growth (≥ 10^4^ CFU/ml) in the urine of healthy pregnant women in rural Burkina Faso. Among 155 *E. coli* isolates obtained from 5934 healthy women, AMR rates were significantly lower compared to *E. coli* isolates obtained from clinical samples (mostly blood cultures) in the same district.

### Comparison with other studies

In the present study, ASB was defined as growth of one species of *Enterobacterales* in counts of 10^5^ CFU/ml or more and was present in 2.1% of women. For antimicrobial susceptibility testing we used the quantitative cut-off of ≥ 10^4^ CFU/ml to define ‘significant growth’, as previously done in an international survey of antimicrobial susceptibility in uncomplicated urinary tract infections [21].

The ASB proportion in the present study was lower compared to some earlier studies from sub-Saharan Africa, citing proportions of 7–40% [17, 22–24], but it was comparable to those reported elsewhere (range 1.9–9.5%) [25, 26]. The presently lower proportions of ASB may be related to the stringent definition, i.e. including only *Enterobacterales* as significant organisms, whereas in most other studies with higher ASB proportions, *Staphylococci* represented a substantial number of cases [17, 22–24].

AMR rates among the *E. coli* isolates obtained from urine of pregnant women were significantly lower compared to AMR rates of clinical isolates. Among the individual antibiotics, this difference was most apparent for ciprofloxacin, i.e. 16.2% for the urine isolates in pregnant women versus over 60% among clinical isolates. Likewise, proportions of ESBL producing and MDR isolates among *E. coli* from urine in healthy pregnant women were 3.2% and 5.2% versus 35.4%, and 60.4% respectively among clinical isolates. It is tempting to speculate that these differences reflect the use of antibiotics such as ciprofloxacin and third generation cephalosporins in the community setting.

The proportion of ESBL producers among the clinical *E. coli* isolates of the comparator studies (35.4%) was slightly lower compared to the 45% reported for sub-Saharan Africa in recent meta-analyses [27–29]. Carriage rates of ESBL producing *E. coli* from stool samples ranged from 38% in Chad to 58% in the Central African Republic [30, 31]. For the urine isolates obtained in pregnant women, AMR rates were lower compared to those found in other cross-sectional studies assessing ASB among pregnant women in sub-Saharan Africa. A study from Ghana from 2018 reported high resistance rates among *E. coli* to nitrofurantoin (35.4%), ciprofloxacin (48.8%), gentamicin (41.5%) and cefuroxime (32.9%) [32]. Two studies from Nigeria (2007 and 2010) reported resistance rates among *E. coli* of approximately 20% against second generation cephalosporins, 40% against gentamicin and 20–70% against ciprofloxacin [22, 33], which was similar to results from a study performed in Uganda in 2010 [34]. A possible explanation for the observed difference to our results is the fact that we strictly excluded participants with symptoms and signs of urinary tract infection. Likewise, the regional and between-country differences may reflect antibiotic use in hospitals as well as in the community, but also in the sectors of animal health and the environment.

### *Escherichia coli* from urine in healthy pregnant women as an indicator of AMR in the community

In the present cohort of over 6000 pregnant women attending ANC, only 1.5% declared symptoms suggestive of an urinary tract infection and only 1.7% had evidence of antibiotic use as demonstrated by urine analysis. The latter proportion is very low compared to 30–40% antibiotic use (based on parents’ declaration) among children suspected of invasive bacterial infection in three of the comparator studies [13–15]. As such, the presently observed low AMR rates among *E. coli* isolates from the urine of healthy pregnant women tends to confirm our pre-study assumption, i.e. there may be a risk of overestimation of AMR rates when performing surveillance on selected clinical samples [35]. However, other factors must be taken into account when comparing resistance rates between both groups. First, the species *E. coli* has distinct pathotypes displaying different degrees in pathogenicity and AMR [36]; further genetic studies are planned to assess the pathotypes of the isolates from pregnant women versus those of the clinical samples. Further, in view of poorly implemented *Infection Prevention & Control* programmes in healthcare facilities, it is not excluded that part of the clinical isolates were belonging to a particular hospital-associated cluster.

Despite the above considerations, it is tempting to forward *E. coli* in urine of pregnant women as a potential indicator for benchmarking, comparing and monitoring community AMR rates across communities over different countries and regions. Such community AMR data generate valuable information about the empiric choice of antibiotics in the local context [37, 38] but may also reflect the effect of AMR control measures. As shown at least in this study setting (and to be confirmed in other settings as well), pregnant women have limited illness and antibiotic use and are accessible through ANC clinics. As part of ANC clinics, urine is routinely sampled for dipstick analysis of glucose and protein and WHO recommends midstream urine culture for the diagnosis of ASB [39]. The dipslide devices presently used were affordable (cost approximately 1 €/device) and user-friendly; they have a long shelf-life (6–9 months at room temperature) and allow for reliable inoculation on-site and subsequent transport to the laboratory. At the downside, there are the challenges of midstream-urine sampling (including contamination) and the reading of the colony counts on the dipstick devices as discussed above. Moreover, the proportion of significant growth is low. Leukocyte esterase and nitrite analysis (incorporated in most urine dipsticks) can be used as a screening tool to select samples for culture (40) but in the present study they were not very accurate to predict growth; further research for a reliable biomarker predicting growth is recommended.

### Limitations and strengths

As noted above, reading of colony counts on dipslide devices tended to be subject to interpretation and this may have impacted the classification of non-significant growth, significant growth and ASB. However, our results showed that pathogen and AMR profiles were similar between *E. coli* from the latter two groups. Second, despite well-designed instructions and training, 8.3% of the samples were contaminated, probably related to the less stringent urine sampling in the context of a ANC compared to clinical care. Third, in retrospect, we realized that the GPA system of parity had not been fully understood by all study nurses, leading to possible too low reported numbers of nullipara compared to primipara. Additionally, there were missing data from 36 consecutive participants (0.6% of all included patients) at one of the healthcare centers. Strengths included the systematic methods used to perform this study, with high numbers of participants included and a consistent work-up by a small team of nurses and laboratory staff. The definitions for ASB and contamination were stringent, adding to the robustness of data.

## Conclusion

In conclusion, in this cross-sectional study among healthy pregnant women attending ANC in rural Burkina Faso, we retrieved significant growth in 3.4% of urine samples, with *E. coli* representing over three-quarters of isolates. AMR rates were considerably lower among these urine samples compared to *E. coli* isolates obtained from clinical isolates in the same study area. Pending further research (geographic generalizability of proportions of growth and predominance of *E. coli*), *E. coli* obtained from urine culture during ANC visits has the potential of an indicator organism for benchmarking and monitoring AMR rates across populations worldwide.

## Supplementary Information

**Additional file 1**.** Table S1**. Overview of demographic data of unique study participants.** Table S2**. Antibiotic resistance among other significant growth obtained from urine samples of healthy pregnant women.** Table S3**. Breakdown of leukocyte esterase and nitrite in relation to significant- and not clinically significant growth.

## Data Availability

All data are available from the researchers upon reasonable request.
