# Supplementary material for: Escherichia coli from urine samples of pregnant women as an indicator for antimicrobial resistance in the community: a field study from rural Burkina Faso
Source: Antimicrob Resist Infect Control. 2022 Sep 5;11:112. doi: 10.1186/s13756-022-01142-7 (PMC9446845; doi:10.1186/s13756-022-01142-7)
Supplement: Supplementary file 1 — Additional file 1. Table S1. Overview of demographic data of unique study participants. Table S2. Antibiotic resistance among other significant growth obtained from urine samples of healthy pregnant women. Table S3. Breakdown of leukocyte esterase and nitrite in relation to significant- and not clinically significant growth. [file 13756_2022_1142_MOESM1_ESM.docx]

| **Additional file 1: Table S1. Overview of demographic data of unique study participants** | | | |
| --- | --- | --- | --- |
| **Participants as stratified by age*** | **< 20 years** | **20-29 years** | **≥30 years** |
|  | n = 1163 | n = 2850 | n = 1728 |
| Age (median [IQR]) | 18 (18 - 19) | 24 (22 - 27) | 33 (30 - 36) |
| Trimester (median [IQR]) | 3 (2 - 3) | 3 (2 - 3) | 3 (2 - 3) |
| Gestation |  |  |  |
| nullipara (% within age group) | 5 (0.4%) | 2 (0.7%) | 0 (0) |
| primipara (% within age group) | 960 (82.6%) | 393 (13.8%) | 14 (0.8%) |
| multipara (% within age group) | 191 (16.4%) | 1922 (67.4%) | 380 (22.0%) |
| grand multipara (% within age group) | 7 (0.6%) | 533 (18.7%) | 1334 (77.2%) |
| Reported antibiotic use prior to sampling (nr. [%]) | 6 (0.5%) | 14 (0.5%) | 0 (0) |

* Among patients with full data available (n = 5741)

| **Additional file 1: Table S2**. Antibiotic resistance among other significant growth obtained from urine samples of healthy pregnant women. | | | |
| --- | --- | --- | --- |
|  |  |  |  |
|  | ***Escherichia coli**** | ***Klebsiella* spp*.*** | **Other *Enterobacterales***** |
|  | **n = 13** | **n = 32** | **n = 12** |
| Ampicillin (n [%]) | 6 (46.2%) | 32 (100%) | 9 (75.0%) |
| Cotrimoxazole (n [%]) | 6 (46.2%) | 11 (34.2%) | 3 (25.0%) |
| Ciprofloxacin (n [%]) | 2 (15.4%) | 2 (6.3%) | 1 (8.3%) |
| Gentamicin (n [%]) | 0 | 0 | 1 (8.3%) |
| ESBL- producing (n [%]) | 0 | 1 (3.2%) | 1 (8.3%)*** |
| MDR (n [%]) | 2 (15.4%) | 2 (6.3%) | 1 (8.3%) |
| MDR + gentamicin (n [%]) | 0 | 0 | 1 (8.3%) |
| ESBL + MDR (n [%]) | 0 | 1 (3.2%) | 1 (8.3%) |
| * *Escherichia coli* growing in mixed flora  *** Enterobacter* spp. (n = 4), *Proteus* spp. (n = 5), *Citrobacter* spp. (n = 3) | | | |
| *** One *Enterobacter* sp. was an ESBL producer with combined resistance to ciprofloxacin, cotrimoxazole and gentamicin. | | | |
|  |  |  |  |

| **Additional file 1: Table S3**. Breakdown of leukocyte esterase and nitrite in relation to significant- and not clinically significant growth | | | | |
| --- | --- | --- | --- | --- |
|  |  |  |  |  |
|  |  | **Leukocyte esterase** | **Nitrite** | **Both** |
|  | **n** | **n (%)** | **n (%)** | **n (%)** |
| **Clinically significant growth** | **202** | **73 (36.1%)** | **28 (13.9%)** | **19 (9.4%)** |
| *Escherichia coli* | 155 | 58 (37.4%) | 25 (16.1%) | 17 (10.9%) |
| *Klebsiella* spp. | 33 | 10 (30.3%) | 3 (9.1%) | 2 (6.1%) |
| Other *Enterobacterales* | 14 | 5 (35.7%) | 0 | 0 |
| **No clinically significant growth** | **3440** | **696 (20.2%)** | **27 (0.8%)** | **13 (0.4%)*** |
| Growth < 10^4^ CFU/ml | 2945 | 562 (16.3%) | 21 (0.7%) | 9 (0.3%) |
| *Enterobacterales* | 52 | 11 (21.2%) | 0 | 0 |
| Other growth | 2893 | 551 (19.2%) | 21 (0.7%) | 9 (0.3%)* |
| *Staphylococcus aureus* | 21 | 10 (47.6%) | 3 (14.3%) | 3 (14.3%) |
| Mixed flora | 146 | 37 (25.3%) | 2 (1.3%) | 0 |
| Skin or environmental contaminants | 321 | 87 (27.1%) | 1 (0.3%) | 1 (0.3%) |
| No identification, or no growth on subculture | 7 | 0 | 0 | 0 |
| Leukocyte esterase was defined as positive in case of a quantification of + or more  Nitrite was classified as positive in case of a quantification of + or more  Percentages represent the percentage of total cases within that growth category which was positive for either leukocyte esterase, nitrite or both  ***** Of whom 1 had taken antibiotics prior to sampling. | | | | |
